# Resolve integer ambiguity based on the global deep grid-based algorithms

**DOI:** 10.1038/s41598-023-47461-6

**Published:** 2023-11-23

**Authors:** Gang Zhi, Junpeng Shi

**Affiliations:** Henan College of Transportation, Zhengzhou, 454052 China

**Keywords:** Aerospace engineering, Space physics, Astronomy and planetary science

## Abstract

Grid theory is rather commonly-used through out the research of integer ambiguity. In order to promote the efficiency of computation, it is of great necessity to reduce the correlations of the grid basis through the reduction. The classical reduction algorithm is known as the LLL (Lenstra–Lenstra–Lovász) algorithm. So as to further enhance the reduction effect, the deep-insertion LLL algorithm can be utilized as an alternative to the basis vector exchange algorithm. In practice, the deep-insertion LLL algorithm can achieve a better reduction effect, but it requires more time for reduction. The PotLLL algorithm replaces the basis vector exchange condition of deep-insertion LLL with an improving in the basis quality, and it can run in polynomial time, but with certain limitations. Therefore, this article proposes a global deep-insertion PLLL algorithm (GS-PLLL) to address the issue of integer ambiguity. GS-PLLL adopts a global strategy for deep-insertion processing, and introduces a rotation sorting method for preconditioning the grid basis. Comparative evaluations were conducted using simulation experiments and real-world measurements on the LLL, DeepLLL, PotLLL, and GS-PLLL algorithms. The experimental results indicate that the GS-PLLL algorithm achieves a better reduction effect than the PotLLL algorithm while improving the efficiency of reduction.

## Introduction

The high-precision positioning equation of the Global Navigation Satellite System (GNSS) can be concluded as the problem of minimizing the following equation subject to constraints.

1 $$ \mathop {\min }\limits_{{{\mathbf{a,b}}}} \left\| {{\mathbf{y}} - {\mathbf{Aa}} - {\mathbf{Bb}}} \right\|_{{{\mathbf{Q}}_{{\mathbf{y}}} }}^{2} \, {\mathbf{a}} \in {\mathbf{Z}}^{n} ,{\mathbf{b}} \in {\mathbf{R}}^{n} $$

In the equation, ${\mathbf{y}}$ represents the double-difference observation value of carrier phase; ${\mathbf{a}}$ is the double-difference ambiguity vector; ${\mathbf{b}}$ is the unknown vector of the baseline after double-difference correction; ${\mathbf{A}}$, ${\mathbf{B}}$ are the design matrices of ambiguity and baseline, respectively; $\left\| . \right\|_{{{\mathbf{Q}}_{y} }}^{2} = \left( . \right)^{*} {\mathbf{Q}}_{y}^{ - 1} \left( . \right)$ and ${\mathbf{Q}}_{y}$ represents the covariance matrix of the double-difference carrier phase observations.

The core of the Eq. (1) lies in whether the integer ambiguity can be resolved in a prompt and accurate manner, which is crucial for the accuracy of the positioning result. Due to the integer constraint of ambiguity ${\mathbf{a}}$ in Eq. (1), cannot be directly solved, so the problem is usually solved based on two steps. Firstly, ignoring the integer property of ${\mathbf{a}}$ and treating it as a real number, Eq. (1) is transformed into an ordinary unconstrained least squares problem, thus obtaining the real-valued estimate ${\hat{\mathbf{a}}}$ of the ambiguity and its corresponding variance–covariance matrix. Then, the corresponding minimization criterion (2) is constructed for the floating-point solution to solve the problem:

2 $$ \arg \min \left( {\left\| {{\hat{\mathbf{a}}} - {\mathbf{a}}} \right\|_{{{\mathbf{Q}}_{{{\hat{\mathbf{a}}}}} }}^{2} } \right) $$

With the continuous development of GNSS technology, solving the problem of integer ambiguity [Eq. (2)] has become a hot topic in the GNSS research field. Among the methods for ambiguity resolution, the LAMBDA (Least Square Ambiguity Correlation Adjustment) algorithm proposed by Teunissen^1^ is the most representative. The LAMBDA algorithm first proposed the concept of decorrelation, which reduces the correlation between vectors and improves the efficiency of ambiguity search by using unitary matrices to process the covariance matrix. Subsequently, many scholars conducted extensive research on the ambiguity and correlation issues and proposed numerous practical solutions. Chang^2^ improved and applied the sorting-based QR decomposition strategy to the LAMBDA algorithm, proposing the MLAMBDA algorithm, which improves the decorrelation and search process of the LAMBDA algorithm. Xu^3^ used the Cholesky decomposition to construct an integer Gaussian matrix and proposed an inverse integer Cholesky decorrelation method based on the pre-sorting strategy. The inverse integer Cholesky decorrelation method outperforms the integer Gaussian decorrelation and LLL algorithms, thus indicating that integer Gaussian decorrelation is not the best decorrelation technique and can be improved further. Chen et al.^4^ used the floating-point transformation matrix to completely decorrelate non-diagonal elements and proposed the Zero-correlation algorithm, experiments have demonstrated that the method can improve the integer resolution efficiency without decreasing the accuracy.

Research has shown that the decorrelation concept in the LAMBDA algorithm is equivalent to the lattice basis reduction processing in lattice theory, and the problem of GNSS whole number ambiguity resolution is equivalent to the closest vector problem (CVP) in lattice theory^5^. Therefore, the problem of integer ambiguity resolution can be approached from the perspective of lattice theory. A Cholesky decomposition is performed on the covariance matrix in the ambiguity solution process in Eq. (2):

3 $$ {\mathbf{Q}}_{{\hat{a}}} = {\mathbf{B}}^{{\text{T}}} {\mathbf{B}} $$

Since the matrix ${\mathbf{Q}}_{{\hat{a}}}$ is a full-rank matrix, the vectors in the decomposed matrix ${\mathbf{B}}$ are independent of each other. Therefore, the objective function in Eq. (2) can be transformed into:

4 $$ \left\| {{\mathbf{a}} - {\hat{\mathbf{a}}}} \right\|_{{{\mathbf{Q}}_{{{\hat{\mathbf{a}}}}} }}^{2} = \left( {{\hat{\mathbf{a}}} - {\mathbf{a}}} \right)^{{\text{T}}} {\mathbf{B}}^{{\text{T}}} {\mathbf{B}}\left( {{\hat{\mathbf{a}}} - {\mathbf{a}}} \right) = \left\| {{\mathbf{y}} - {\mathbf{Ba}}} \right\|^{2} $$

In the equation, ${\mathbf{y}} = {\mathbf{B}}\hat{\mathbf{{a}}}$. Based on the knowledge of lattice theory, if we consider the matrix ${\mathbf{B}}$ as the basis matrix, and each vector in the matrix as the lattice basis, then an n-dimensional full-rank lattice can be constructed.

5 $$ L({\mathbf{B}}) = \left\{ {{\mathbf{l}}\left| {{\mathbf{l}} = \sum\limits_{i = 1}^{n} {z_{i} {\mathbf{b}}_{i} ,z_{i} \in Z} } \right.} \right\} $$

Further equivalence of Eq. (4) is achieved through the structure of the lattice:

6 $$ \left\| {{\mathbf{y}} - {\mathbf{Ba}}} \right\|^{2} \le \left\| {{\mathbf{y}} - {\mathbf{l}}} \right\|^{2} ,\forall {\mathbf{l}} \in {\mathbf{L}}({\mathbf{B}}) $$

Equation (6) represents the CVP problem in lattices. There is no controversy about the use of lattice theory for solving integer ambiguities because the core idea of the LAMBDA algorithm, which is currently widely used for solving ambiguities, is referenced to lattice theory. The quality of the lattice basis is crucial for solving the CVP problem. Even when using the same CVP algorithm, the computational efficiency and the accuracy of the results may vary greatly depending on the lattice basis used. The development of lattice basis reduction techniques has greatly contributed to the resolution of the CVP problem. Utilizing lattice basis reduction, the original lattice can be simplified, resulting in a smaller and more efficiently computable basis. This leads to faster computation of the optimal solution to the Closest Vector Problem (CVP). The study of lattice basis reduction algorithms originated from Gauss and Lagrange's work on quadratic homogeneous equaion^6^. Korkine and Zolotareff^7^ extended the Gauss reduction algorithm to any dimensionality and proposed the KZ reduction algorithm to solve the shortest vector problem in lattices. Lenstra et al.^8^ proposed the LLL reduction algorithm, which can solve the shortest vector problem in polynomial time. Hassibi et al.^9^ first used the LLL reduction algorithm in the field of GNSS integer ambiguity resolution, it is shown that the integer least squares problem associated with parameter estimation can be solved efficiently in practice. Grafarend et al.^10^ provided a detailed analysis of the principles of the LLL reduction algorithm for solving integer ambiguities in GNSS. Liu et al.^11^ proposed an improved algorithm based on matrix-wide rounding to address the rounding error in LLL. Fan^12^ used a block Gram-Schmidt orthogonalization algorithm to improve the average correlation coefficient and condition number and thus enhance the efficiency of the LLL algorithm. Lu^13^ used block dimensionality reduction to improve the LLL algorithm and effectively increase its operational efficiency. Xie et al.^14^ improved the LLL reduction algorithm by using Householder orthogonal transformation based on system rotation to reduce the basis vector length. Through experimental analysis, Householder LLL (HLLL) algorithm can significantly improve the effect of correlation reduction processing. However, the algorithm still has the shortcoming of long statute time, which needs to be further improved. Recently Li et al.^15^ made further advancements to the HLLL by using column selection to obtain higher reduction efficiency and superior reduction performance. Schnorr proposed the DeepLLL reduction algorithm based on the idea of deep insertion^16^. Although DeepLLL has better output quality than LLL in practical applications, the complexity of DeepLLL may be super-exponential. Fontein et al.^17^ proposed a new variant of DeepLLL called PotLLL, which has a provable polynomial runtime by replacing the basis vector exchange condition of DeepLLL with an improvement in the quality of the basis matrix.

However, both DeepLLL and its variant PotLLL only operate on the sublattice $\mathcal{L}_{k}$ generated by a subset $\left( {{\mathbf{b}}_{1} , \ldots ,{\mathbf{b}}_{k} } \right)$ at each iteration. This means that based on the condition of exchanging base vectors, only ${\mathbf{b}}_{k}$ can deep insertion and swapped to $\left( {{\mathbf{b}}_{1} , \ldots ,{\mathbf{b}}_{k - 1} } \right)$, which limits the choice of deep insertion within the sub-lattice $\mathcal{L}_{k}$ during iteration. This paper proposes a global deep insertion strategy to improve PtoLLL. When performing deep insertion swaps, it is no longer restricted to the current index, but instead selects a pair of indices $\left( {i,k} \right) \,$ globally on the entire lattice to deeply insert ${\mathbf{b}}_{k}$ into position $i$. Deep insertion can be performed as long as the potential after reordering is reduced by at least some increment $\left( {1 - \delta } \right)$ compared to the potential before sorting. When the algorithm terminates, the output potential of the base cannot be further reduced by deep insertion. Furthermore, considering the impact of covariance matrix sorting on the efficiency of ambiguity resolution, a rotation sorting algorithm is used to pre-sort the lattice basis to further improve the efficiency of the lattice basis reduction algorithm. The lattice basis reduction algorithm proposed in this paper, which combines the global deep insertion strategy and the pre-sorting strategy, is called GS-PLLL.

The rest of this paper consists of the following sections. “integer ambiguity resolution based on lattice theory” provides a description of LLL and introduces the DeepLLL and PtoLLL algorithms. “GS-PLLL lattice basis reduction algorithm” describes the two main improvement parts of the GS-PLLL algorithm: greedy global deep insertion and rotation sorting. “Results and discussion” presents experimental results by comparing the performance of the proposed algorithms to LLL and various parallel algorithms. Finally, the “Conclusion” section provides a summary and conclusions.

## Integer ambiguity resolution based on lattice theory

### LLL lattice basis reduction

The LLL algorithm is a reduction algorithm with groundbreaking significance in the field of lattice basis reduction proposed jointly by three scholars, Lenstra, Lenstra, and Lovasz. The basic idea of this algorithm is to gradually map the basis vectors to a set of more independent vectors that are closer to the standard orthogonal basis through a series of basis transformations (Gram–Schmidt orthogonalization and basis shortening). Perform Schmidt orthogonalization on the upper triangular matrix B in (3):

7 $$ {\mathbf{B}} = {\mathbf{B}}^{*} \times {\mathbf{O}} = \left[ {{\mathbf{b}}_{1}^{*} , \cdots ,{\mathbf{b}}_{i}^{*} , \cdots ,{\mathbf{b}}_{n}^{*} } \right] \times \left[ {\begin{array}{*{20}c} 1 & \cdots & {o_{1k} } & \cdots & {o_{1n} } \\ {} & \ddots & \vdots & {} & \vdots \\ {} & {} & 1 & \cdots & {o_{kn} } \\ {} & {} & {} & \ddots & \vdots \\ {} & {} & {} & {} & 1 \\ \end{array} } \right] $$

where ${\mathbf{B}}^{*} = \left[ {{\mathbf{b}}_{1}^{*} , \cdots ,{\mathbf{b}}_{i}^{*} , \cdots ,{\mathbf{b}}_{n}^{*} } \right]$ is an orthogonal matrix corresponding to the matrix ${\mathbf{B}}$ and ${\mathbf{b}}_{i}^{*} = {\mathbf{b}}_{i} - \sum\limits_{j = 1}^{i - 1} {o_{ji} {\mathbf{b}}_{j}^{*} ,i > j}$; ${\mathbf{O}}$ is an upper triangular orthogonal transformation matrix and satisfies $o_{ji} = \left\langle {{\mathbf{b}}_{i} ,{\mathbf{b}}_{j}^{*} } \right\rangle /\left\| {{\mathbf{b}}_{j}^{*} } \right\|_{2}^{2}$. If the following conditions are satisfied for matrices ${\mathbf{B}}$, ${\mathbf{B}}^{*}$, and ${\mathbf{O}}$:

i. $$\left| {o_{i,j} } \right| \le 0.5{ , }1 \le j < i \le n$$ (Size reduction).
ii. $\delta \left\| {{\mathbf{b}}_{k - 1}^{*} } \right\|^{2} \le \left\| {\pi_{k - 1} \left( {{\mathbf{b}}_{k} } \right)} \right\|^{2}$, where $\pi_{k - 1} ({\mathbf{b}}_{k} ) = {\mathbf{b}}_{k}^{*} + r_{k,k - 1} {\mathbf{b}}_{k - 1}^{*}$ (Exchange basis vectors according to the Lovász condition).

The lattice basis ${\mathbf{B}}$ is called an LLL-reduced basis, where the parameter $\delta \in \left( {0.25,1} \right]$ is a pre-selected parameter, which is usually set to 0.75 when solving the integer ambiguity problem.

When converting between different bases of the same lattice, it can be done using unimodular matrices. If the orthogonal transformation matrix ${\mathbf{O}}$ in the Schmidt orthogonalization satisfies the unimodular matrix conditions, then the orthogonal matrix ${\mathbf{B}}$ obtained through orthogonalization can also be used as the basis of this lattice. However, this situation occurs very rarely. Therefore, when reducing the lattice basis, it is not possible to achieve complete orthogonality between vectors like in GS orthogonalization. The only goal is to make the basis vectors as orthogonal as possible while satisfying the condition of unimodular transformations.

Lattice basis reduction requires that all elements of the unimodular matrix must be integers and the absolute value of the determinant must be 1. To meet these requirements, rounding must be used to standardize the orthogonalization coefficients. Under the constraint of scaling reduction, complete integer orthogonal transformations can be achieved between vectors, resulting in a good lattice basis reduction effect. The effect of lattice basis reduction depends on the magnitude of the orthogonalization coefficients, which are related to the dot product of the two vectors and the lengths of the orthogonalized vectors. The coefficient for orthogonalizing a subsequent vector is inversely proportional to the length of the orthogonalized vector corresponding to the previous vector. If the longer orthogonalized vector is arranged first, the coefficient for computing the subsequent vector will be smaller, and may even become 0 after rounding. At this point, it is considered that the angle between the two vectors is greater than 60 degrees, and good orthogonalization has been achieved. According to the cosine formula for the angle between two vectors, the cosine of the angle between vectors depends on the lengths of the two vectors. The calculation of the orthogonalization coefficient replaces two vectors with one vector. If the length of this vector is large, the calculated cosine value will be small and cannot reflect the true angle between the two vectors. Therefore, while constraining the orthogonalization coefficients, constraints must also be placed on the lengths of the vectors.

8 $$ \left\| {{\mathbf{b}}_{1}^{*} } \right\|^{2} \le \left\| {{\mathbf{b}}_{2}^{*} } \right\|^{2} \le \cdots \le \left\| {{\mathbf{b}}_{n}^{*} } \right\|^{2} $$

Due to the restrictions of unimodular matrices, it is difficult to achieve the goal of LLL-reduced basis. The LLL algorithm analyses the projection vector lengths of two adjacent vectors in the subspace spanned by the earlier basis vectors to ensure that the length of the first basis vector is the shortest, that is, the basis vector exchange condition.

To summarize, the process of LLL reduction transformation based on the matrix B and O obtained from the Schmidt orthogonal transformation of the lattice basis is as follows:

#### Magnitude reduction process

The orthogonalization coefficient reflects the angle between two vectors. The larger the value, the smaller the angle. Therefore, LLL reduction performs a magnitude reduction through an integer transformation, which makes the orthogonalization coefficient of each vector satisfy the scaling reduction condition. For the basis vector ${\mathbf{b}}_{i}$, its corresponding orthogonalization coefficient is $o_{j,i} = \frac{{\left\langle {{\mathbf{b}}_{i} ,{\mathbf{b}}_{j}^{*} } \right\rangle }}{{\left\langle {{\mathbf{b}}_{j}^{*} ,{\mathbf{b}}_{j}^{*} } \right\rangle }}$. According to the requirements of unimodular matrices, the following integer transformation matrix can be constructed:

9 $$ {\mathbf{T}}_{j,i} = {\mathbf{I}} - \left[ {o_{j,i} } \right]_{round} {\mathbf{e}}_{j} {\mathbf{e}}_{i}^{T} $$

where ${\mathbf{I}}$ is the identity matrix, and ${\mathbf{e}}$ is the unit vector. Equation (9) is used to transform the basis vectors and orthogonalization coefficient matrix to satisfy the magnitude reduction condition of the LLL reduction algorithm.

#### Length reduction process

This process aims to ensure that the length of the orthogonalized vectors meets the length reduction condition. It is implemented simultaneously with the magnitude reduction process by changing the order of the vectors ${\mathbf{b}}_{i}$ and ${\mathbf{b}}_{i + 1}$ to satisfy the basis vector exchange condition. The exchange of adjacent vectors will cause changes in the orthogonalization coefficients and orthogonalized vectors. Therefore, after performing length reduction, the orthogonalization coefficients and vectors must be updated.

### Deep insert LLL lattice basis reduction algorithm

According to the exchange condition of basis vectors in the LLL algorithm, only the adjacent basis vectors ${\mathbf{b}}_{i}$ and ${\mathbf{b}}_{i + 1}$ can be exchanged. This leads to a large number of basis vector exchanges during the reduction process and weak exchange effects, which directly affect the final reduction efficiency and effectiveness. To solve this problem, the DeepLLL reduction algorithm extends the exchange condition of basis vectors in the LLL reduction algorithm, allowing for the exchange of non-adjacent basis vectors. For lattice bases ${\mathbf{B}}$, ${\mathbf{B}}^{*}$, and ${\mathbf{O}}$, the basis vector exchange condition of the LLL reduction algorithm is changed to the deep insertion exchange condition: for $1 \le i < k \le n$, if $\left\| {\pi_{i} \left( {{\mathbf{b}}_{k} } \right)} \right\|^{2} \ge \delta \left\| {{\mathbf{b}}_{i}^{*} } \right\|^{2}$ is satisfied, then the lattice basis ${\mathbf{B}}$ is called a Deep-LLL reduction basis. It should be noted that when $i = k - 1$, the deep insertion exchange is equivalent to the basis vector exchange condition in the LLL reduction algorithm.

### PotLLL lattice basis reduction algorithm

For a lattice basis ${\mathbf{B}} = \left( {{\mathbf{b}}_{{\mathbf{1}}} ,{\mathbf{b}}_{2} , \cdots ,{\mathbf{b}}_{n} } \right)$, the potential is defined as follows:

10 $$ Pot({\mathbf{B}}): = \prod\limits_{i = 1}^{n} {vol\left( {\mathcal{L}_{i} } \right)^{2} } = \prod\limits_{i = 1}^{n} {\left\| {{\mathbf{b}}_{i}^{*} } \right\|^{{2\left( {n - i + 1} \right)}} } $$

The advantage of using the potential of a lattice basis is that it takes into account not only the number of vectors in the lattice basis, but also the influence of the arrangement order of the basis vectors. The contribution of earlier basis vectors to the value of Pot(B) is significantly greater than that of later basis vectors. Since PotLLL involves the order relationship between basis vectors, the following concepts related to basis vector sorting are introduced.

Let $S_{n}$ be a permutation of n elements. For $\sigma \in S_{n}$ and a lattice basis ${\mathbf{B}}$, $\sigma \left( {\mathbf{B}} \right) = \left( {{\mathbf{b}}_{\sigma \left( 1 \right)} , \ldots ,{\mathbf{b}}_{\sigma \left( n \right)} } \right)$ is defined as the ordering of the basis vectors, where $\sigma \left( j \right)$ represents the $j$th position of the sorted basis $\sigma \left( {\mathbf{B}} \right)$. The permutation $\sigma_{i,k} \in S_{n} \, ,{ 1} \le {\text{i < k}} \le {\text{n}}$ used subsequently is defined as follows:

11 $$ \sigma_{i,i} \left( j \right) = \left\{ {\begin{array}{ll} j & \quad if \, j < i \, or \, k < j \\ k &\quad if \, j = i \\ j - 1 &\quad if \, i + 1 \le j \le k \\ \end{array} } \right. $$

Among them, ${\mathbf{b}}_{k}$ is inserted between ${\mathbf{b}}_{i - 1}$ and ${\mathbf{b}}_{i}$, and the $i$-th to $k - 1$-th vectors are moved one position forward, while the other vectors remain in place. Like DeepLLL, PotLLL only changes the exchange condition of the basis vectors in LLL. If the lattice basis vectors are all satisfied by $\delta \cdot P\left( {\mathbf{B}} \right) \le P\left( {\sigma_{i,k} {\mathbf{B}}} \right)$ for $1 \le i < k \le n$, they are called PotLLL-reduced basis.

## GS-PLLL lattice basis reduction algorithm

The basis exchange condition in the DeepLLL algorithm can help the lattice basis to meet the ascending order requirement to some extent. However, the deep-insert exchange condition limits the exchange to be only within the current sublattice, which may result in too many exchanges and negatively affect the reduction efficiency and quality of the reduced basis. To solve this problem, a pre-sorting strategy can be adopted to reduce the number of basis exchanges^18^, or a new evaluation criterion can be used to improve the basis exchange condition and enhance the quality of the reduced basis. The quality of the reduced basis is closely related to the decoding time of the ambiguity, and a higher quality reduced basis means a higher search efficiency. In this paper, a GS-PLLL lattice basis reduction algorithm is proposed based on matrix pre-sorting and global deep-insertion exchange, aiming to improve both reduction efficiency and the quality of the reduced basis.

### Rotation sort

The rotation sorting method was initially proposed by Xu et al.^19^ based on Gaussian decomposition. Its main idea is to rotate the smallest conditionally variance element through the sorting matrix to the first element position in the matrix during each sorting. The rotation sorting method based on lattice requires improvement from the perspective of lattice basis reduction. Assuming the first rotation sorting matrix is ${\mathbf{P}}_{1}$, we have:

12 $$ {\mathbf{P}}_{1} {\mathbf{Q}}_{{\hat{a}}} {\mathbf{P}}_{1}^{{\text{T}}} = \left[ {\begin{array}{*{20}c} {q_{\min } } & q \\ {q^{{\text{T}}} } & {{\tilde{\mathbf{Q}}}_{{\hat{a}}} } \\ \end{array} } \right] $$

where ${\tilde{\mathbf{Q}}}_{{\hat{a}}}$ is the submatrix obtained by removing the first row and first column of ${\mathbf{Q}}_{{\hat{a}}}$; $q_{\min }$ is the minimum element on the diagonal of the covariance matrix. After arranging the smallest element on the diagonal of ${\mathbf{Q}}_{{\hat{a}}}$ to the position of the first element, QR decomposition is performed on the lattice basis to obtain ${\mathbf{B}}^{*}$ and ${\mathbf{O}}$. Finally, lattice basis construction and further QR decomposition are performed on the sorted submatrix ${\tilde{\mathbf{Q}}}_{{\hat{a}}}$. By iteratively applying the rotation sorting to the submatrices, the lattice basis can meet the ascending order requirement even before the lattice basis reduction algorithm is applied.

### Greedy global deep insertion

LLL, DeepLLL, and PotLLL all use linearly increasing or decreasing basis vector indices for k in the range of $2 \le k \le n$ when reducing the lattice basis, and they only perform the reduction on the sublattice $\mathcal{L}_{k}$ generated by the first $k$ basis vectors of the lattice basis ${\mathbf{B}}$. The key innovation of the algorithm based on the global deep-insertion strategy is that it applies to the entire lattice basis, without restricting the deep-insertion to any sublattice $\mathcal{L}_{k}$. Moreover, the deep-insertion ${\mathbf{B}} \leftarrow \sigma_{i,k} \left( {\mathbf{B}} \right)$ closely follows the scaling reduction to ensure the scaling reduction of the whole basis, and to prepare for the next iteration.

In addition to processing the entire lattice basis during basis vector exchange, a greedy strategy is also used to select an index $\left( {i,k} \right) \,$ to minimize the potential of the lattice basis at each iteration in the GS-PLLL algorithm. By selecting the pair of indices that leads to the largest potential drop in the lattice basis at each iteration, the GS-PLLL algorithm can rapidly meet the criteria for PotLLL reduction.

**Figure 1:**
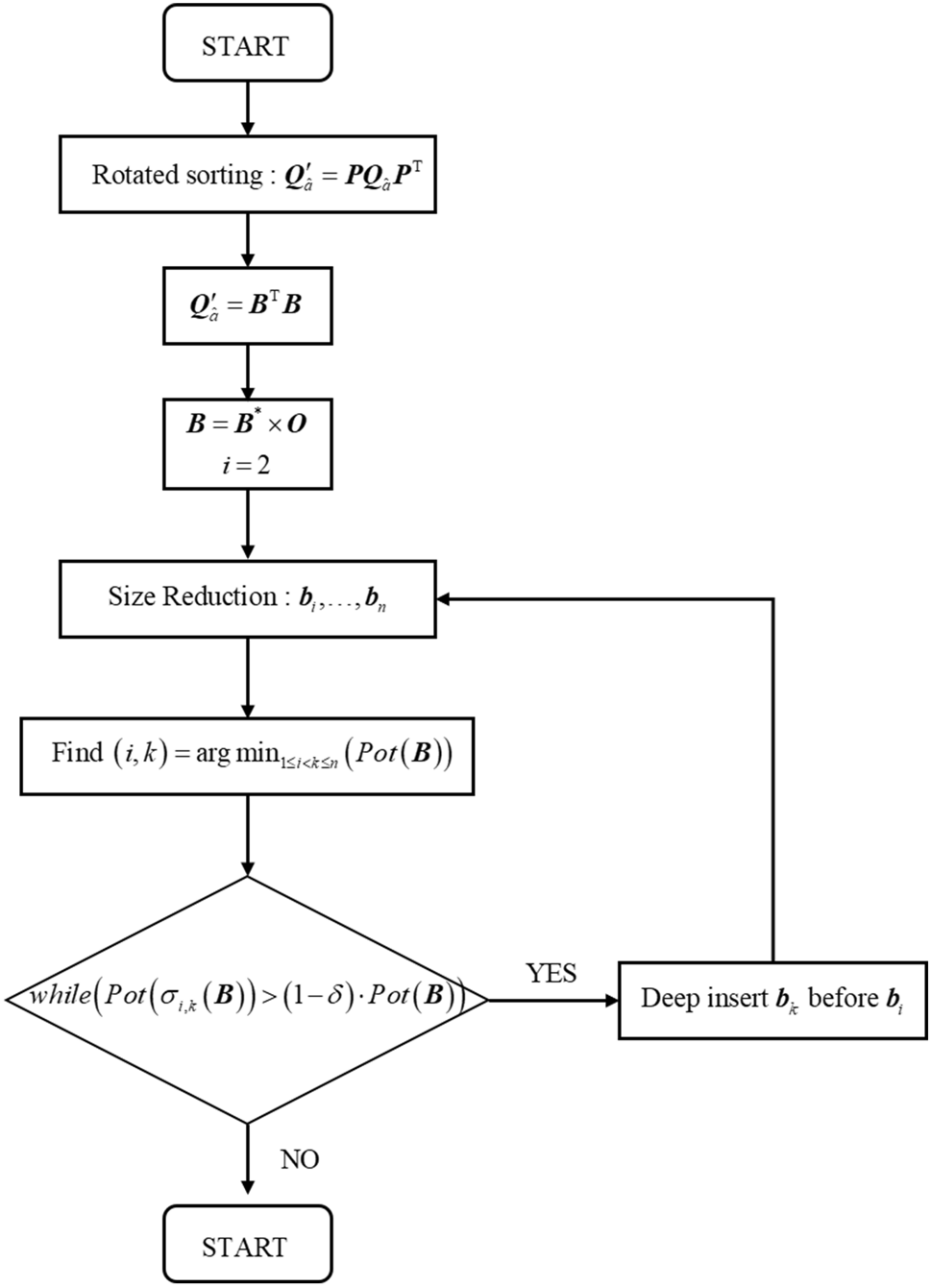
The algorithmic flow of GS-PLLL.

In summary, the algorithmic flow of GS-PLLL, combined with rotation sorting and greedy deep insertion, is shown in Fig. 1.

## Results and discussion

To evaluate the performance of the GS-PLLL reduction algorithm and compare it with the LLL, DeepLLL, and PotLLL reduction algorithms, both simulated data and actual measurement data were used. The simulated data provides a general representation unaffected by external factors, allowing for a better illustration of the advantages of the three lattice basis reduction algorithms. The simulation experiments verified the theoretical advantages of the GS-PLLL algorithm, while the actual measurement data further validated its effectiveness. All experiments were conducted on a computer platform with Windows 10 operating system, using an Intel® Core™ i7-8750H CPU @ 2.20Hz with 16GB memory.

The goal of lattice basis reduction is to transform the basis vectors of a lattice to make them shorter and more orthogonal, thus simplifying computations and improving efficiency^20^. In order to measure the performance of lattice basis reduction, the Hadamard ratio is often used as an evaluation index^21^. The Hadamard ratio is used to describe the degree of orthogonality of a set of vectors, with a range of $\left( {0,1]} \right.$. The closer two vectors are to being orthogonal, the closer their Hadamard ratio is to 1, and the reciprocal of the Hadamard ratio is called the orthogonality defect. The Hadamard ratio of a lattice basis ${\mathbf{B}} = \left( {{\mathbf{b}}_{{\mathbf{1}}} ,{\mathbf{b}}_{2} , \cdots ,{\mathbf{b}}_{n} } \right)$ is defined as follows:

13 $$ H({\mathbf{B}}) = \left( {\frac{{\det {\mathbf{L}}}}{{\left\| {v_{1} } \right\|\left\| {v_{2} } \right\| \cdots \left\| {v_{n} } \right\|}}} \right)^{1/n} $$

In addition, the number of basis vector exchanges directly affects the efficiency of basis reduction algorithms. Therefore, the superiority of the GS-PLLL reduction algorithm is explained by comparing the number of basis vector exchanges, computational efficiency, and the Hadamard ratio.

### Experiment 1

In Experiment 1, the random matrix construction method proposed by Chang et al.^2^ was used, with the covariance matrix of the integer ambiguity constructed using a random simulation method. In order to prove the universality of the algorithm, the simulated random matrix had dimensions ranging from 5 to 40, covering the low- to high-dimensional cases of the covariance matrix. Additionally, in order to avoid accidental situations, each experiment was conducted with 100 repetitions to obtain the mean value.

**Figure 2:**
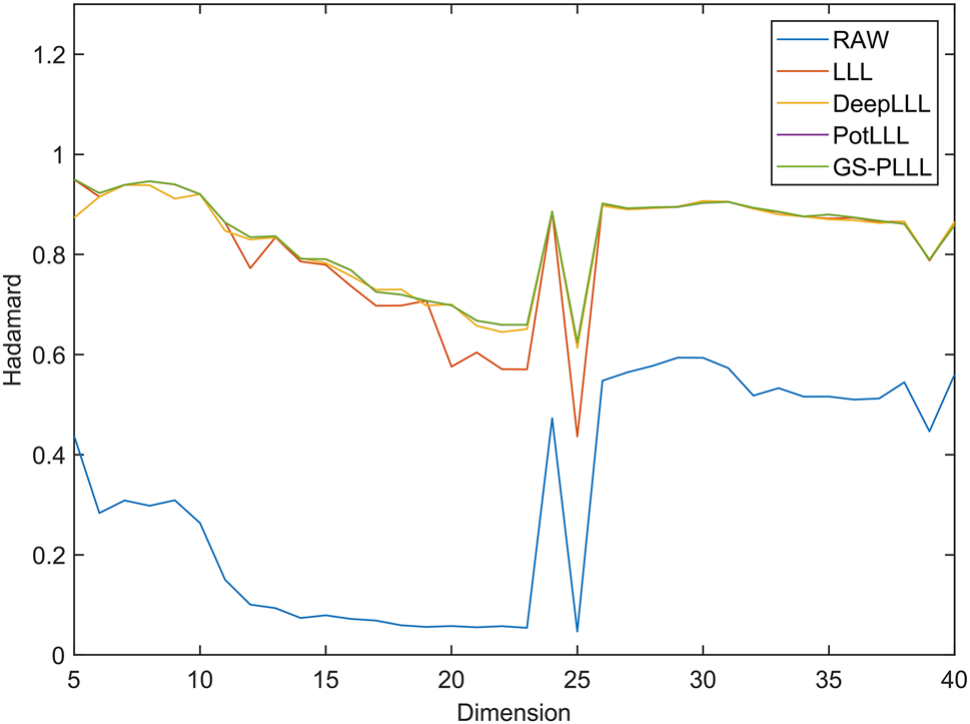
The distribution graph of Hadamard ratios for Experiment 1.

Figure 2 provides a preliminary comparison of the Hadamard ratios of the LLL, DeepLLL, PotLLL and GS-PLLL algorithms on the original data. Given the random nature of matrix construction, the subsequently generated Hadamard ratios are irregular random numbers. This property is evident when examining the folding of the Hadamard ratios in the original matrix, as shown in Fig. 2. The figure emphasises that each of the four algorithms effectively enhances the Hadamard ratios, although the GS-PLLL algorithm shows very little change from the results of the PotLLL algorithm. This is primarily because the GS-PLLL algorithm employs rotation sorting and greedy global depth interpolation to enhance PLLL. While these approaches significantly bolster the reduction's efficiency, their contribution to the overall quality improvement of the reduction is relatively modest.

For a more visual comparison, Table 1 presents the statistical results of the Hadamard ratios obtained from the LLL, DeepLLL, PotLLL, and GS-PLLL algorithms.

**Table 1:** Comparison of reduction effect in Experiment 1.

	LLL	DeepLLL	PotLLL	GS-PLLL
Max	0.9498	0.9389	0.9498	0.9501
Min	0.4364	0.6133	0.6244	0.6247
Mean	0.8139	0.8280	0.8340	0.8343

The table clearly demonstrates that the GS-PLLL algorithm achieves the highest restoration quality in terms of the Hadamard ratio. Combining the findings from Table 1, it can be concluded that the GS-PLLL algorithm exhibits a slightly superior reduction effect compared to the PotLLL algorithm. This observation aligns with the results reported by Fontein et al.^17^, which indicated a higher improvement rate for the PotLLL algorithm when compared to DeepLLL and LLL techniques.

Overall, the results presented in Table 1 and Fig. 2 confirm that the GS-PLLL algorithm outperforms the other algorithms, highlighting its superior restoration quality in terms of the Hadamard ratio. These findings reinforce the significance and effectiveness of the GS-PLLL algorithm in integer ambiguity resolution for high-dimensional GNSS positioning.

**Figure 3:**
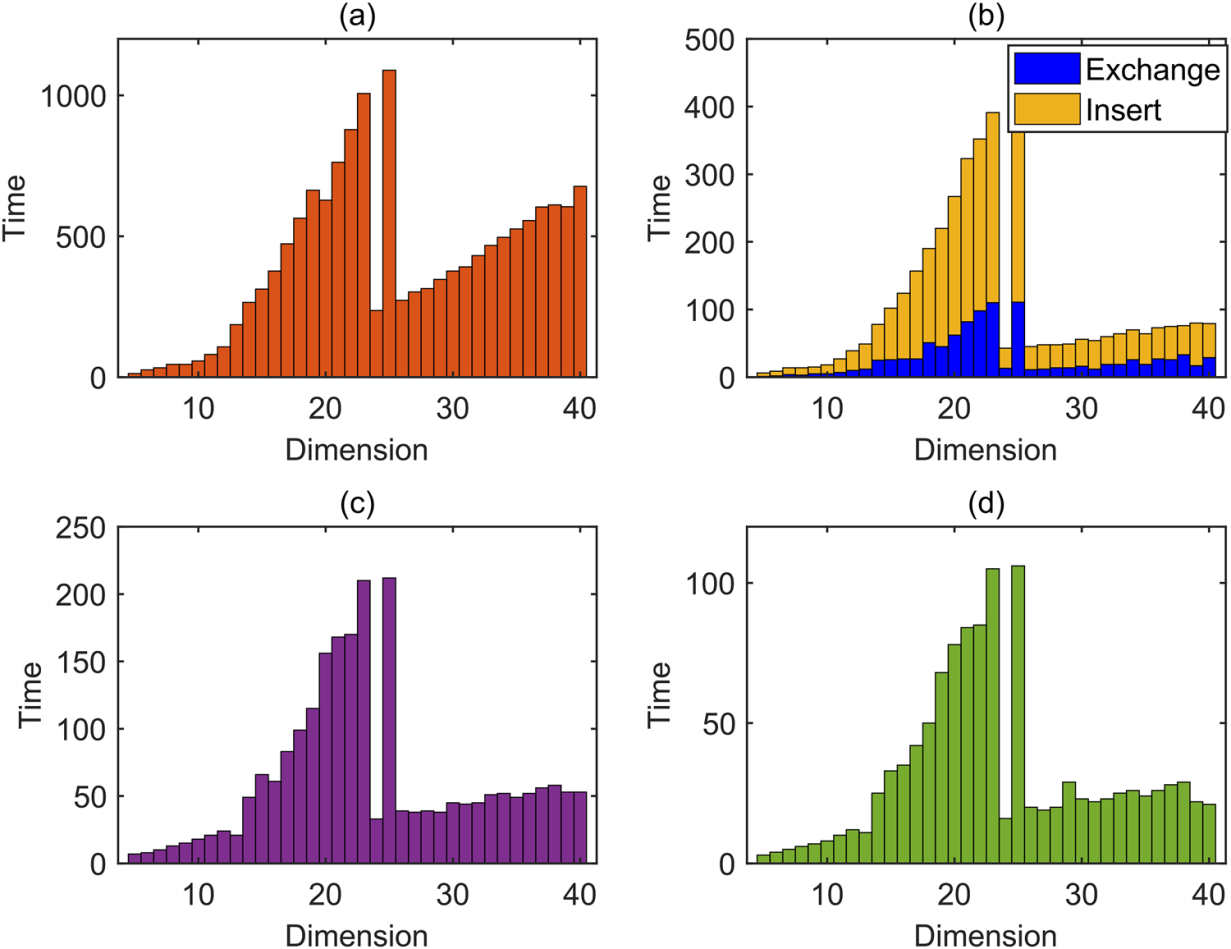
The comparison graph of the number of basis vector exchanges in Experiment 1.

In lattice basis reduction algorithms, scaling reduction and basis vector exchange directly impact the efficiency of ambiguity resolution. Among them, the impact of scaling reduction on efficiency improvement is limited, whereas basis vector exchange directly affects the resolution efficiency^22^. For a lattice basis reduction algorithm, the fewer the number of base vector exchanges, the higher the reduction efficiency. Therefore, Fig. 3 compares the number of base vector exchanges among four lattice basis reduction algorithms. In the figure, (a), (b), (c), and (d) respectively represent the LLL, DeepLLL, PotLLL, and GS-PLLL algorithms. As the DeepLLL algorithm divides the base vector exchange steps into deep insertion steps and adjacent vector exchange steps, Fig. 3b displays a stacked bar chart of these two steps in the DeepLLL algorithm. From the Fig. 3, it can be seen that compared to the LLL algorithm, DeepLLL, PotLLL, and GS-PLLL algorithms all reduce the number of basis vector exchanges, and among the four algorithms, the GS-PLLL algorithm has the fewest exchanges. It is worth noting that in these four algorithms, we can observe that the number of exchanges at 25 dimensions far exceeds that at 40 dimensions. The reason is that the number of exchanges in the lattice basis reduction algorithm is related to the matrix dimension and the correlation of the initial matrix. For example, in the case of 25 dimensions, due to the poor correlation between the vectors of the original matrix, the Hadamard ratio is only 0.0469, so when using the three algorithms for calculation, they exchanged 1089 times, 391 times, 210 times, and 105 times respectively. In the case of 40 dimensions, the Hadamard ratio of the initial matrix is 0.5603, indicating good correlation. Therefore, although the dimension is larger, the number of exchanges for the three algorithms is less than in 25 dimensions, specifically 677 times, 79 times, 53 times, and 21 times.

Overall, the results in Fig. 3 highlight the effectiveness of the GS-PLLL algorithm in achieving minimum basis vector exchange, demonstrating its superior efficiency in solving integer ambiguity.

**Figure 4:**
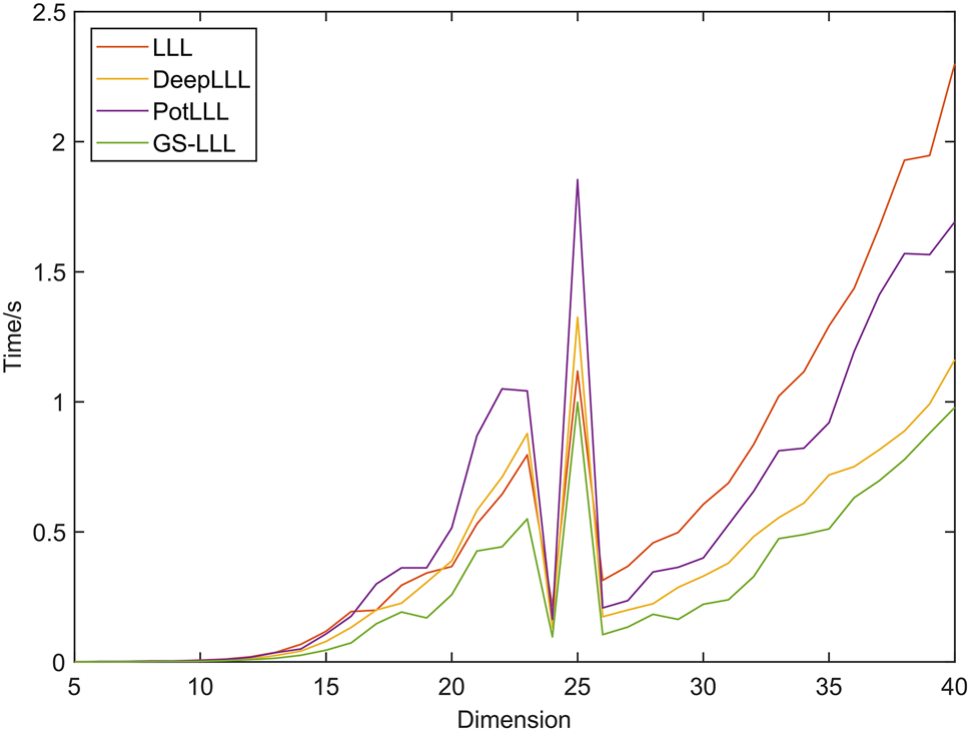
Experiment 1 time comparison chart.

To further highlight the advantages of the GS-PLLL algorithm, a comparison of the computation times of the four algorithms is presented in Fig. 4. The figure reveals important insights regarding their performance.

Based on the comparison data from Fig. 4, it can be seen that the PLLL algorithm performs poorly when processing low-dimensional original matrices, especially those with high correlation. Additionally, when processing high-dimensional original matrices, particularly those with low correlation, the computation time of the PLLL algorithm also exceeds that of the LLL algorithm. This is because, in situations where the original matrix has poor correlation, the PLLL algorithm requires a large number of deep interpolation exchanges. However, the GS-PLLL algorithm overcomes these drawbacks of the PLLL algorithm and demonstrates the highest computational efficiency in all scenarios. These findings indicate that the GS-PLLL algorithm outperforms other algorithms in terms of both reduced computation times and improved reduction efficiency.

Furthermore, it can be observed that in the case of 25 dimensions, the computation time of all four algorithms significantly increased. The reason for this phenomenon is that the low correlation of the original 25-dimensional matrix led to a substantial increase in the number of basis vector exchanges. Combining the conclusions from Fig. 3, we can infer that the computation time for lattice basis reduction is influenced not only by the correlation of the original matrix but also by the dimension of the matrix.

### Experiment 2

To further validate the performance of the GS-PLLL reduction algorithm in real-world scenarios, after verifying the theoretical advantages of GS-PLLL in experiment1, actual measured data was used for further validation. The experimental procedure involved the use of actual measurement data collected by Huace P5 satellite differential receivers. The base station and mobile station observations were recorded in the RINEX 3.02 format, with an observable elevation angle cutoff of 5° and a sampling rate of 1 Hz. Precise ephemeris information was obtained from the Satellite Navigation and Positioning Research Center of Wuhan University. The L1 frequency signals from GPS satellites are used in this experiment for double-difference modelling to compute the ambiguity floating-point solutions of the double-difference observations and their corresponding covariance matrices. Like simulation experiments, actual measurement experiments also discuss three aspects: Hadamard ratios, the number of base vector exchanges, and computational efficiency.

**Figure 5:**
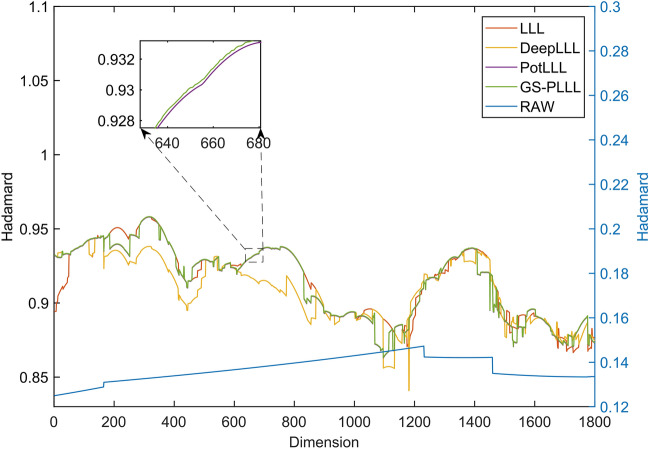
Experimental comparison chart of Hadamard in Experiment 2.

Figure 5 illustrates the comparison among the Hadamard ratios of LLL, DeepLLL, PotLLL, and GS-PLLL algorithms concerning the Hadamard ratios of the original matrices. The left y-axis represents the Hadamard ratios of the four studied algorithms, while the right y-axis denotes the Hadamard ratios of the raw data. It is evident from the figure that all four algorithms achieve satisfactory performance in terms of effectiveness. However, it is worth noting that the DeepLLL algorithm exhibits relatively unstable effectiveness, likely due to the weak constraints of its conditions. Moreover, as observed in the inset graph, the PotLLL algorithm ranks slightly below the GS-PLLL algorithm but still outperforms the DeepLLL and LLL algorithms in general, aligning with the conclusion from Experiment 1. These results further substantiate the superiority of the GS-PLLL algorithm in terms of effectiveness.

**Figure 6:**
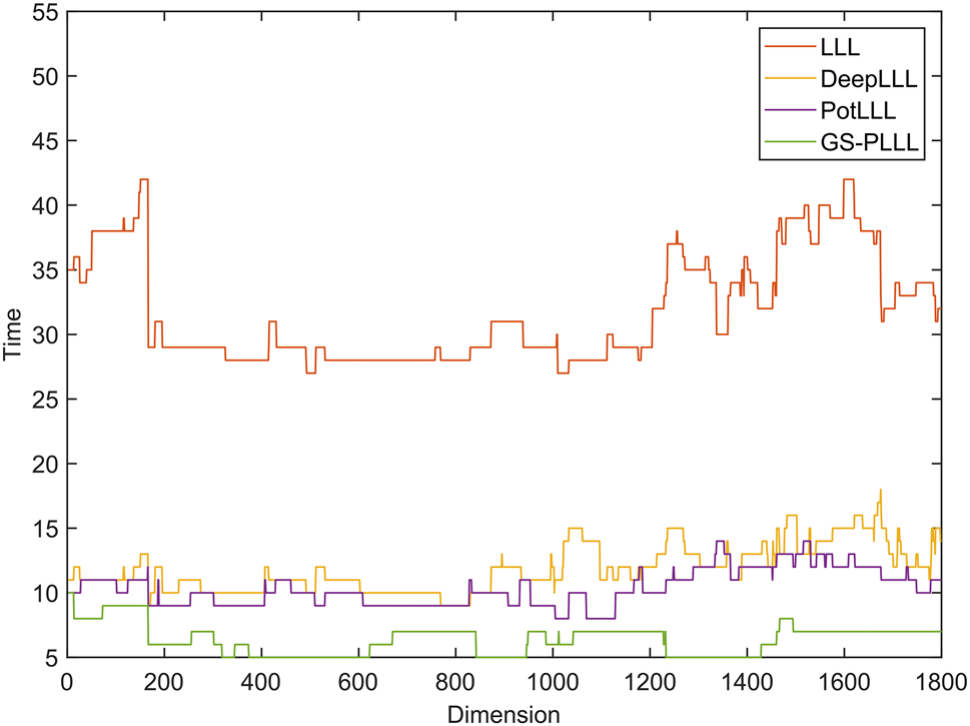
Experimental chart of the number of basis vector exchanges in Experiment 2.

**Figure 7:**
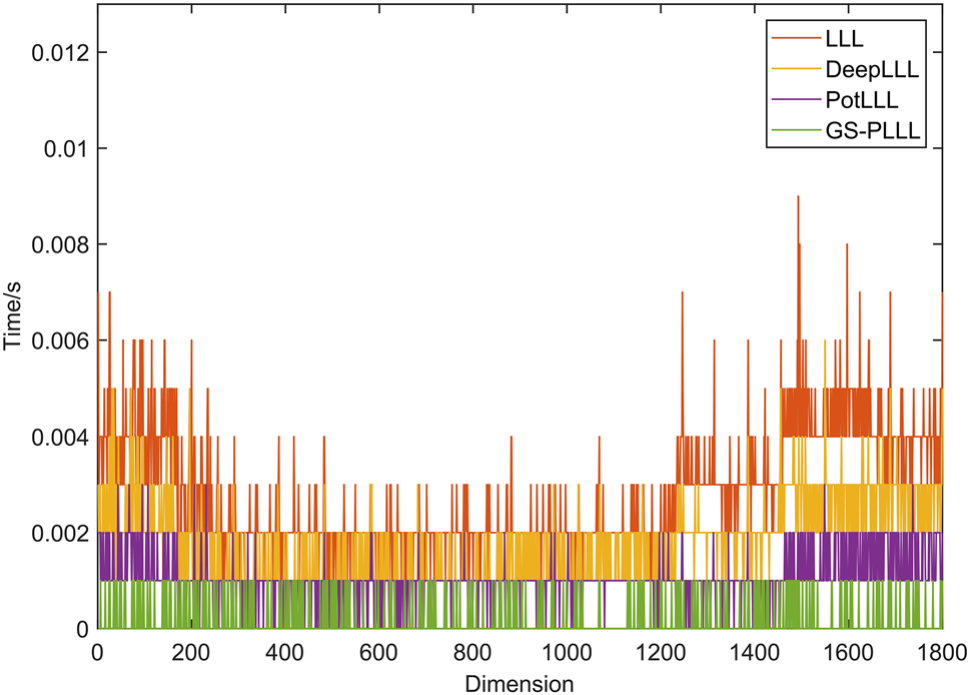
Experimental comparison chart of computation times in Experiment 2.

As illustrated in Fig. 6, the GS-PLLL algorithm significantly minimizes the quantity of necessary basis vector exchanges for the reduction. According to statistical analyses, the LLL algorithm averages 32 basis vector exchanges, compared to the 12 of DeepLLL, the 10 of PotLLL, and the mere 6 of GS-PLLL. This noticeable reduction arises from the global deep interpolation measurement of GS-PLLL, which effectively decreases the number of base vector exchanges. It's important to note that the quantity of base vector exchanges directly impacts the reduction efficiency of the algorithm: fewer exchanges correspond to higher efficiency. Hence, theoretically, the GS-PLLL algorithm possesses the highest reduction efficiency. Figure 7 aims to further corroborate this assertion by comparing the computation times of the LLL, DeepLLL, PotLLL, and GS-PLLL algorithms.

Figure 7 provides additional evidence for the superior reduction performance of the GS-PLLL algorithm. The figure clearly shows that the GS-PLLL algorithm exhibits the shortest computation time among the compared algorithms. This can be attributed to the implementation of the global deep insertion and rotation sorting strategies in the GS-PLLL algorithm, which significantly enhances the efficiency of lattice basis reduction.

## Conclusions

In this paper, a new reduction algorithm, GS-PLLL, is proposed to solve the integer ambiguity problem in high-dimensional GNSS positioning. The algorithm incorporates global depth insertion and rotation sorting strategies. To validate the feasibility of GS-PLLL and evaluate its performance, we compare GS-PLLL with other algorithms (i.e., LLL, DeepLLL, and PotLLL) using simulated and real-world measurement data.

The evaluation was based on various metrics, including the Hadamard ratio, the number of basis vector exchanges, and computation times. The experimental findings consistently demonstrated the superiority of the GS-PLLL algorithm. Compared to the other algorithms, it exhibited a shorter reduction time and achieved a higher Hadamard ratio, indicating its low computational complexity and ability to generate better reduction basis. Besides, the statistical analysis of computation times revealed that the GS-PLLL algorithm outperformed the other algorithms in speed and stability. Accordingly, it is concluded that the GS-PLLL algorithm is an effective and practical approach for fast integer ambiguity resolution in high-dimensional GNSS positioning.

In addition, how to borrow the idea of heuristics algorithms^23–25^ to improve the computational efficiency of lattice-based reduction is also a direction for future research.

### Supplementary Information

Supplementary Information.

## Data Availability

The authors confirm that the data supporting the findings of this study are available within the article and its Supplementary materials. The Supplementary materials include simulation data and raw data of the experiments. Details on how to access these materials can be found in the documentation. The datasets analysed in this study are managed by Henan College of Transportation and are available upon request by contacting the corresponding author. The precise ephemeris dataset used in this study can be downloaded from the IGS Data Center of Wuhan University at http://www.igs.gnsswhu.cn/. All the raw data used are in standard RINEX format, including ephemeris data and observation data.
