# Supplementary material for: Resolve integer ambiguity based on the global deep grid-based algorithms
Source: Sci Rep. 2023 Nov 23;13:20597. doi: 10.1038/s41598-023-47461-6 (PMC10667259; doi:10.1038/s41598-023-47461-6)
Supplement: Supplementary file 1 — Supplementary Information. [file 41598_2023_47461_MOESM1_ESM.zip › supplement materials of the manuscript/Document description.doc]

Data Description

The supplementary materials including simulation data and raw data of the experiments. The data of all images are presented in excel files, each image corresponds to an Excel file. The simulated data of the simulation experiments can be viewed in the Randomly Simulated Data.xlsx file. The measured experimental data are in the Measured experimental data folder. All measured experimental data conform to the GNSS standard data format, i.e., RINEX format. A description of the format can be found in the RINEX302 file in the Measured experimental data folder.
